# Subduction thermal regime, petrological metamorphism and seismicity under the Mariana arc

**DOI:** 10.1038/s41598-023-29004-1

**Published:** 2023-02-02

**Authors:** Rui Qu, Weiling Zhu, Yingfeng Ji, Chaodi Xie, Deng Zeng, Fan Zhang

**Affiliations:** 1 State Key Laboratory of Tibetan Plateau Earth System, Environment and Resources (TPESER), Institute of Tibetan Plateau Research, Chinese Academy of Sciences, Beijing, 100101 China; 2 University of Chinese Academy of Sciences, Beijing, 100049 China; 3 Geophysics Department, School of Earth Sciences, Yunnan University, Kunming, 650091 China; 4 Key Laboratory of Ocean and Marginal Sea Geology, South China Sea Institute of Oceanology, Innovation Academy of South China Sea Ecology and Environmental Engineering, Chinese Academy of Sciences, Guangzhou, 510301 China; 5 Southern Marine Science and Engineering Guangdong Laboratory, Guangzhou, 511458 China

**Keywords:** Geophysics, Geodynamics

## Abstract

Because of the steep subduction of a highly concave slab, researchers have characterized megathrusts under the Marianas as among the coldest and curviest plate coupling interfaces in various circum-Pacific subduction zones. Seismic tomography indicates that the heterogeneous underlying plate varies markedly in its subduction angle, velocity, and flexure along the strike and dip, while their effects on the thermal structure and intraslab earthquake occurrence remain enigmatic. By incorporating the 3-D MORVEL velocity and state-of-the-art slab geometry into thermomechanical modeling, we estimated the 3-D subduction thermal state and hydrothermal regime below the Marianas. We find that (1) the concave slab geometry and the complexity of the intraslab velocity variation in the Marianas are associated with a heterogeneous along-strike thermal regime and a cold mantle wedge beneath the central Marianas; (2) amphibolitization and eclogitization of subducted oceanic crust cause variations in fluid pressure and fluid release from the subduction interface, which may influence the distribution of interface seismicity in the Mariana system; (3) the concentration of active hydrothermal vents in the trench > 8 km deep is accompanied by a large temperature gradient and subsequent remarkable slab dehydration in the southern Marianas; and (4) slab dehydration (> 0.02 wt%/km) from 30 to 80 km indicates notable fluid release and potential fluid migration in subduction channels, which may correspond to the large water flux at depth beneath the Marianas.

## Introduction

**Figure 1:**
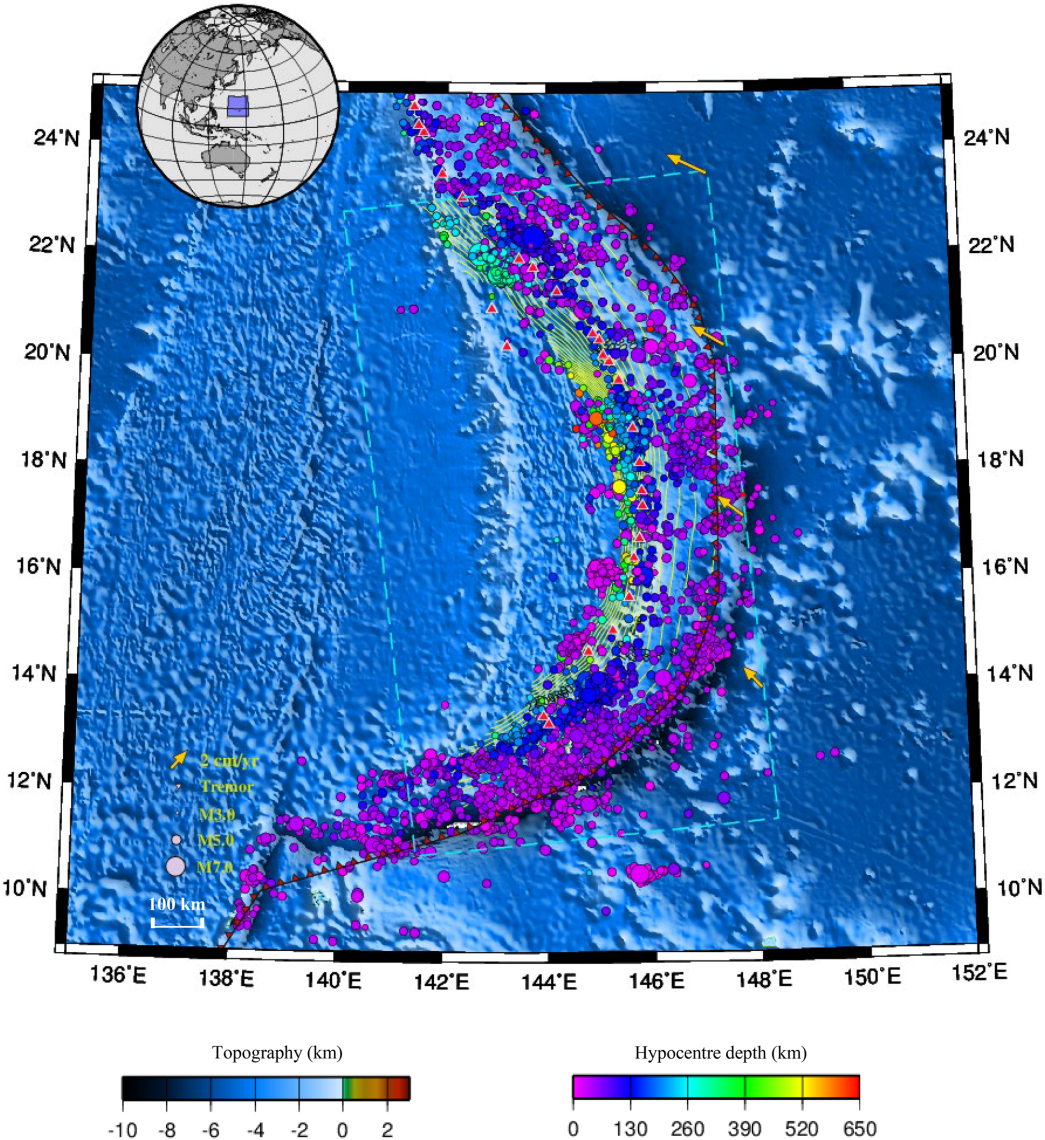
Tectonic map of the Marianas. Background colors indicate the surface topography (ETOPO^19^). Green curved lines indicate the isodepth contours on the upper surface of the Pacific plate with an interval of 20 km (Slab2^20^). Dashed light blue lines show the model region for the subducted Pacific Plate. Red triangles indicate active volcanoes^21^. The black curve with red barbs marks the convergent plate boundary^22^. Colored spheres indicate regular interplate earthquakes that occurred from January 1, 2001, to December 31, 2009 (IRIS^23^). Yellow arrows indicate the motion of the Pacific Plate toward the Philippine Sea Plate (MORVEL^24,25^). The map was created by using the Generic Mapping Tools (GMT)^26^ (version: GMT 4.5.7, URL link: https://www.generic-mapping-tools.org/download/).

**Figure 2:**
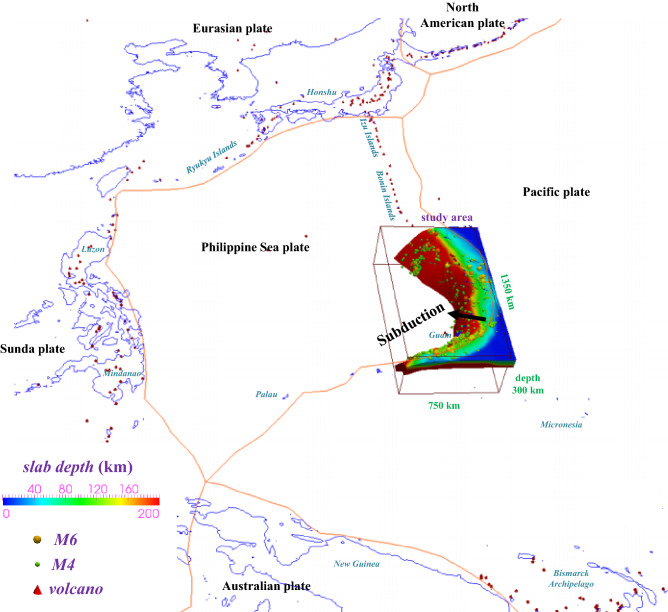
Model domain in the tectonic setting, including the model size, plate boundaries and arc volcanism. The plate boundaries follow Bird (2003)^22^. Red cones indicate active arc volcanoes^21^. The figure was created by using the software Paraview (version: Paraview 5.4.1, URL link: https://www.paraview.org/download/).

The Izu‒Bonin–Mariana (IBM) arc system represents an interoceanic convergent margin, where one of the oldest seafloor regions on the planet has been subducting since 43 Ma^1^ (Figs. 1 and 2). This system experienced two stages of back-arc spreading, forming the Parece Basin between the Palau–Kyushu Ridge and western Mariana Ridge (30–15 Ma) and the Mariana Trough between the western Mariana Ridge and the Mariana arc (7 Ma-present)^2^. Active back-arc extension driven by asthenospheric upwelling marks one of the most significant features of the Mariana subduction zone that is caused by steep subduction^3^. The various slab morphologies that have been further studied in numerical models imply that trench rollback is usually associated with the balance of the force resulting from slab stagnation or penetration in subduction dynamics^4,5^.

Seismogenic megathrusts below the Mariana have constrained the seismicity to less than M8^6,7^. The recurrence of M6 earthquakes in the Mariana is several per year, and most of them are intermediate–deep earthquakes with depths of > 100 km. Historical M7 earthquakes have struck the Mariana as few as once per decade, and the 2016 M7.7 earthquake (212 km depth) 60 km SSE of Agrihan and 2007 M7.5 earthquake (261 km depth) 280 km NW of Farallon de Pajaros have occurred in recent decades. In general, M < 5 earthquakes are more frequently observed and geographically adjacent to the southern Marianas than to the central and northern portions (Fig. 1). This phenomenon probably reflects the differences in fault properties, which are determined by geophysical factors, such as the convergence rate and obliquity, plate dip, age, and geometry^8^. Subduction seismicity is thus likely controlled by the subduction thermal structure or slab tears^9–11^. For example, Kong et al.^8^ found that deep events (at depths of $>$ 300 km) in the northern and southern Mariana arc are potentially affected by a horizontally propagating tear (within a depth range of 300–400 km) that is parallel to the trench. Researchers have proposed that the heterogeneous concave slab is key to an increase in intraslab compression^12^, causing extensional brittle failure^13^ and resulting in various pathways for seawater to hydrate the subducted crust^14^. Slab dehydration thereby contributes to mantle melting and arc magmatism^15^, which also controls energy and fluid budgets for subduction output^16–18^.

Recent studies have shown that along-trench variations in the relative subduction velocity could affect the slab thermal state by altering the effective convergence rate of the underlying oceanic plate toward the overriding plate^27,28^. Current global tectonic motions (MORVEL^24,25^) indicate that the old Pacific seafloor is subducting obliquely (northwestward) into the Mariana margin (Figs. 1 and 2). However, the effects of oblique subduction on such a concave slab thermal structure are complex, and the temperature variation along the megathrust remains enigmatic. Therefore, combined with previous research in seismogenic subduction zones, including southwestern and central Japan, Cascadia, Hikurangi, Chile, Sumatra, Alaska, and Izu‒Bonin^29–38^, we focus on the Mariana and attempt to account for plate motion factors in thermal modeling to evaluate the thermal state of this specific subduction channel underlain by a cold and old oceanic lithosphere and shed more light on the concave subduction system, which remains poorly understood in terms of the relationship between slab metamorphism and seismotectonics regarding oblique and steep subduction.

## Methods and models

**Figure 3:**
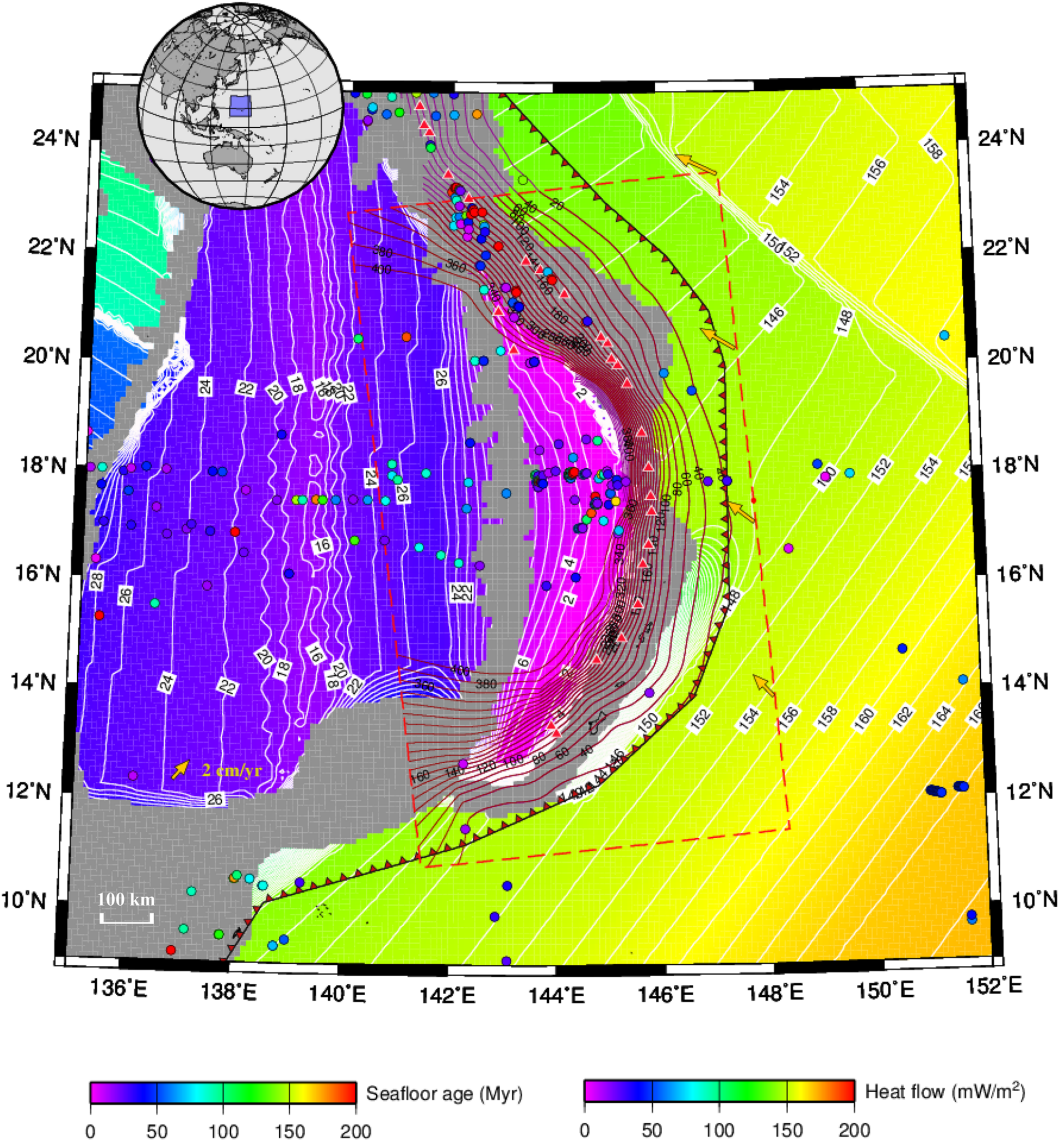
Seafloor age and heat flow in the Marianas. Solid circles represent observations from the global heat flow database^42^. The seafloor ages are from EarthByte^40^. The map was created by using the software GMT^26^ (version: GMT 4.5.7, URL link: https://www.generic-mapping-tools.org/download/).

Previous thermal models developed from code Stag3D and using the finite difference method (FDM)^39^ have been successfully applied to circum-Pacific subduction zones involving northeastern Japan, Cascadia, Hikurangi, and north–central Chile^28–31,34,35^. To continue this study, we perform 3-D, time-evolving thermomechanical modeling for the Mariana, which has dimensions of 1350 × 750 × 400 km (along-strike length × cross-arc width × depth) and 80 × 80 × 100 grids, and we simulate the subduction of the Pacific Plate with varying seafloor ages (Fig. 3). We adopt a model involving a kinematic slab (geometrically prescribed and temporally evolving in length and thickness) and the dynamic domains for the mantle, overriding crust and accretionary prism. The subduction velocities inside a prescribed 3-D constrained volume of the oceanic lithosphere are given at each timestep according to the increase in the slab length, and we set the subduction time to at least 20 Myr to ensure that the model reaches a steady thermal state, with a temperature variation of < 10℃ over time and a lapse time of $\ge$ 5 Myr. Hence, our model differs from a fully dynamic model in which the subducted plate evolves freely. The topography of the incoming plate adopts the Slab2 global data^20^ extrapolated by the surface function of the Generic Mapping Tools for the deep portions of the slab. The oceanic lithosphere has an initial thickness based on spatially distributed seafloor ages provided by EarthByte^40^. The trenchward temperature boundary is assumed according to the plate cooling model^41^ and the plate ages from EarthByte^40^.

**Figure 4:**
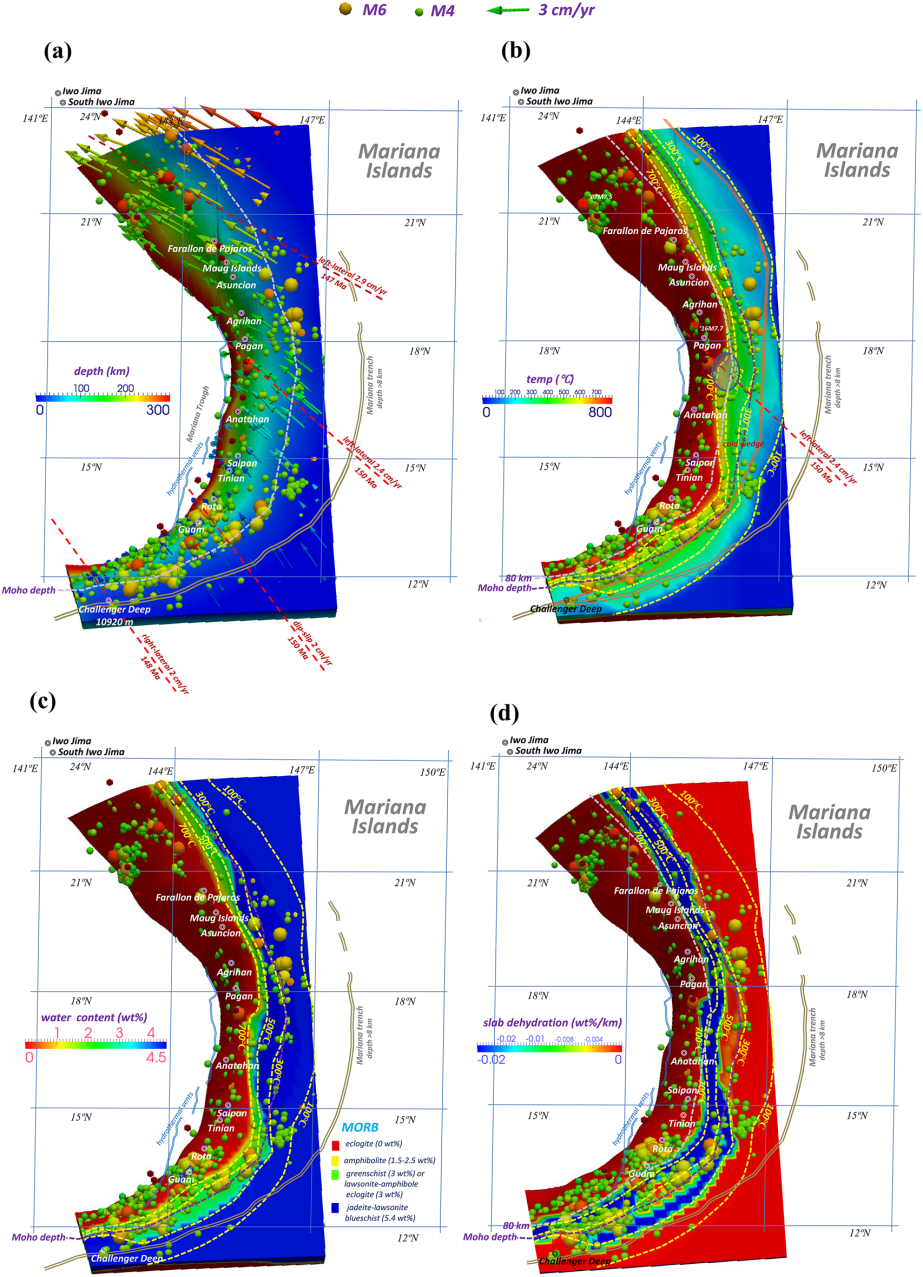
(**a**) Subduction velocity and plate age of the concave slab beneath the Marianas. Earthquake magnitudes are indicated by the colored spheres. Gray double lines represent the Mariana Trench, with a water depth > 8 km (Center for Coastal and Ocean Mapping/Joint Hydrographic, University of New Hampshire). Blue double lines indicate the back-arc hydrothermal vents aligned in the Mariana Trough^47^. Four red dashed lines represent the profiles along the subduction direction and the ages of the trenchward seafloor. (**b**) Calculated thermal state of the plate interface. The yellow dashed lines represent the isotherm contours of 100 °C, 300 °C, 500 °C and 700 °C. Red cones indicate active volcanoes observed from above. The purple dashed line marks the Moho discontinuity. Colored spheres represent earthquakes, as described above. The orange curve indicates the plate boundary^22^. The blue ellipse shows the cold mantle wedge close to Anatahan Island. (**c**) Water content (wt%) in the upper surface of the incoming plate. Yellow dashed lines represent the isotherm contours of 100 °C, 300 °C, 500 °C and 700 °C. Colored spheres indicate earthquakes, as described above. Red cones mark active volcanoes. The purple dashed line indicates the Moho discontinuity. (**d**) Slab dehydration (wt%/km) along the upper surface of the incoming plate. Yellow dashed lines represent the isotherm contours of 100 °C, 300 °C, 500 °C and 700 °C. The figure was created by using the software Paraview (version: Paraview 5.4.1, URL link: https://www.paraview.org/download/).

Surface heat flow observations, including the global heat flow database^42^ and heat flow from the Curie point depth estimates^43^, are employed in this study to further constrain the thermal modeling (Fig. S1). The observed surface heat flow averages < 60 mW/m^2^ in the Mariana forearc basin, which has no accretionary complex but does have an outer-rise high, indicating probable low-density serpentinized mantle lithosphere beneath the outer forearc^44^. Similar to previous thermal models applied to northeastern and SW Japan, Hikurangi, Cascadia, and Chile^28–33^, the subduction velocities are obtained from the MORVEL 25-plate calculator for relative motion angular velocities^24,25^ and extrapolated into the subducted slab (Fig. 4a). The grid cells on the subducted Pacific Plate are prescribed with various motion rates and directions with regard to the Philippine Sea Plate, constituting the kinematic slab domain. The model boundaries and bottom are assumed to be adiabatic and permeable, except that the top boundary is set as rigid and has a temperature of 0 °C. The viscosity for wet olivine follows those from Hirth and Kohlstedt^45^ and Burkett and Billen^46^ for the upper mantle. The composite model settings, including the model configuration, initial and boundary conditions, and physical parameters, are illustrated in the Supplemental Information (SI). Based on these mature model settings, the 3-D thermal state and the phase transition of subducted rocks in the structured tectonic complex in the Marianas become calculable, and the thermal structural features caused by this rare high curvature and steep dip can be further estimated.

## Results

### 3-D subduction velocity field and thermal regime

**Figure 5:**
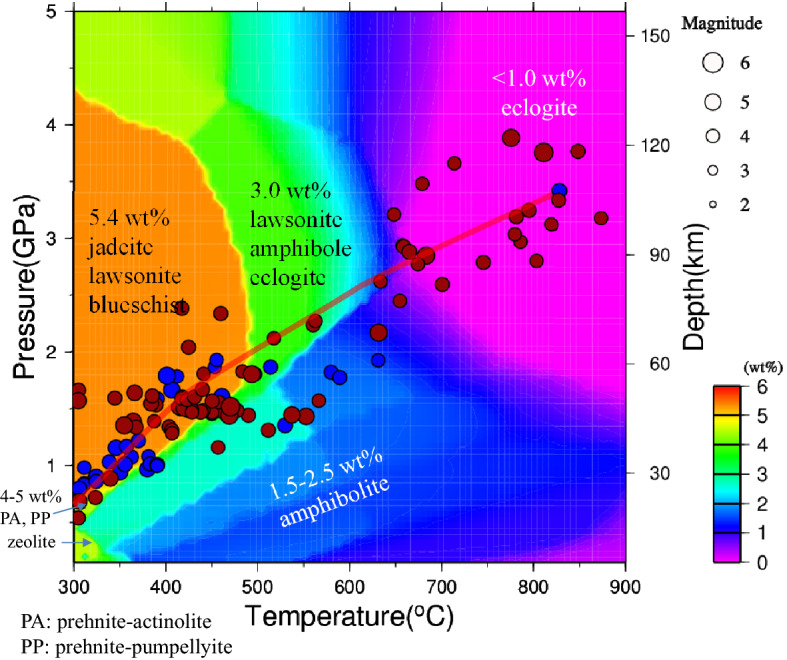
P–T conditions for M > 4 earthquakes near the plate interface plotted on the phase diagram for the metamorphic rocks of subducted oceanic crust (MORB layer) beneath the northern Marianas (blue, < 17°N) and southern Marianas (dark red, > 17°N). Colored circles indicate the interplate earthquakes and intraslab earthquakes, which occurred from Jan. 1, 2000, to Dec. 31, 2009 (IRIS). The figure was created by using the software GMT^26^ (version: GMT 4.5.7, URL link: https://www.generic-mapping-tools.org/download/).

The unstable megathrust is characterized by northwestward strike-slip motion in the northern part and southwestward strike-slip motion in the southernmost part. The oldest age of the incoming Pacific plate is Late Jurassic (> 145 Ma), and the plate is therefore colder than those in most of the other circum-Pacific subduction zones (Fig. 4b). The combination of plate motions, concave morphology, and varying dip angles and slab ages results in a comparatively unique subduction thermal structure that presently enables predictions from a 3D perspective, as Figs. 4 and 5 show. Although the along-strike seafloor ages do not differ much (140–150 Ma), the plate subduction velocity increases from 2 cm/yr for the southern seafloor (Challenger Deep), with an age of 148 Ma, to 2.4 cm/yr at 18°N (Pagan), with an age of 150 Ma, and then to 2.9 cm/yr in the northern Mariana close to Farallon de Pajaros Island, with an age of 147 Ma (Fig. 4a). We note that the largest dip angle is present in the Challenger Deep, where there is right-lateral strike-slip motion, i.e., westward oblique subduction. Straight subduction occurs along the profile in the northern Guam area, with a subduction velocity of 2 cm/yr. Nevertheless, the fastest relative plate motion is in the northernmost portion of the Mariana arc, with a velocity range of 2.9–3.0 cm/yr.

The complex concave subduction system leads to a comparatively complicated thermal regime, as exhibited in Fig. 4b. The calculated thermal regime clearly exhibits a thrust zone that transitions from 300 to 500 °C between the volcanic arc and trench. The coldest mantle wedge in the Marianas is predicted to be located to the east of Anatahan Island in the central Mariana (16–18° N, 146° E, blue circle in Fig. 4b), where the temperature is estimated to be less than 300 °C at approximately 30 km. In the northern Marianas (> 18° N), the Moho interface temperature increases to 400–500 °C along the strike. In the southern counterpart portion, the slab dip becomes larger, and the Moho interface temperature increases to 500 °C, which may be attributed to the decrease in the subduction velocity from 2.4 to 2 cm/yr. At a depth of > 80 km below the Marianas, the slab temperature rapidly increases to more than 700 °C, which is attributable to the stress and yield from slab bending^48^ and the transition to a large dip at this depth. The subvolcanic interface temperature is more than 1000 °C on average. The average P–T conditions for the subduction interface are shown in Fig. 5 (red curve), characterized by a slowdown in the temperature increase at a depth range of 40–60 km, thereby delineating a boundary between the cold shallow and warm deep slab portions.

Furthermore, we consider the P–T conditions at the epicenters of megathrust seismic events beneath the Marianas from 2000 to 2010^23^ and correlate them to the P–T conditions of the subducted mid-ocean ridge basalt (MORB) rocks at the same depth (Fig. 5). The red circles represent earthquakes in the northern Marianas, and the blue circles represent earthquakes in the southern Marianas. We find that the southern events are preferentially distributed on the shallow plate interface (< 60 km), with a pressure of < 2 GPa and a temperature of < 700 °C. These results can also be explained by the slab dehydration rate of the Marianas, which is larger in the south than in the north (Fig. 4d).

### Water content and slab dehydration

Estimates of the water content distribution in the Pacific Plate beneath the Marianas are shown in Fig. 4c based on the phase diagram for mid-ocean ridge basalts^49,50^. Lawsonite blueschist with a water content of 5.4 wt% transforms to lawsonite–amphibole eclogite (3 wt%), greenschist (3 wt%), or amphibolite (1.5–2.5 wt%) at temperatures of approximately 300–500 °C, followed by eclogite (0 wt%) at temperatures of approximately 650–700 °C (Fig. 5). Between 15 and 20°N, amphibolitization occurs mainly ca. 500 °C (depth of 60 km), which is attributable to the presence of a cold mantle wedge and a moderate dip angle at depth (Fig. 4c). In the southern Marianas along the megathrust, amphibolitization is associated with lower temperatures than in the northern Marianas because of a high dip angle, which accelerates the rock phase transition to amphibolite at approximately 400–500 °C and 1.5 GPa. Eclogitization requires comparatively stable transition temperatures of 650–700 °C; thus, the eclogitization front does not change much along the strike (Fig. 4c).

The dehydration belt on the plate interface beneath the Marianas extends from north to south and is aligned offshore from the Marianas Islands, with widths of 80 km (> 20° N), 50 km (16°–20° N), and 100 km (< 16° N) (Fig. 4d). The broader width of the dehydration front in the south is attributed to comparatively flat subduction adjacent to Guam (12°–14° N). Note that the mantle wedge in the southern Marianas between 12 and 14°N obviously accommodates more regular seismicity than that in other portions, partly due to high slab dehydration accompanied by straight subduction. The mantle wedge tends to be a tectonically active region sandwiched between the rigid overriding crust and a high-density, high-pressure viscous upper mantle. Upwelling fluids and melts are prone to be retained at the corner and serpentinize dry forearc crustal rocks^51^. The coldest mantle wedge close to the Anatahan–Pagan Islands (16–18°N) has a thinner dehydration region and is thus less seismically active. The Moho discontinuity at the plate interface in the vicinity of Anatahan is approximately 50 km east of the dehydration front, possibly resulting in fewer earthquakes between 16 and 18° N. In the northern Marianas, this interpretation is complicated because slab MORB dehydration facilitates the occurrence of only shallow interplate earthquakes, while deep subvolcanic earthquakes at depths of > 100 km are supposedly affected by other factors, such as the harzburgitization front in the lower layer of the incoming plate, similar to the situation for deep seismicity in the southern Marianas.

## Discussion

### Subduction regime, surface expression, and arc magmatism

The Mariana volcanic arc ranges from 13 to 20° N above the plate interface, where temperatures are greater than 700 °C, possibly indicating that the dominant control is from the interior oceanic lithosphere, including harzburgitization and the stress and yield from bending^48^. Arc volcanoes located above the cold mantle wedge of the central Mariana arc (Pagan and Agrigan) are associated with interplate temperatures that are 100–200 °C lower between 18 and 19° N, which are immediately downdip othe coldest mantle wedge at 16–18° N and undergo remarkable left-lateral strike-slip (Fig. 4b).

The southern Mariana arc-trench system is rapidly deforming, resulting in unusual interactions between arc and back-arc basin magmatic systems^15^. The back-arc hydrothermal vents that are distributed along the Mariana Trough to the west of the Mariana Island arc^47,52,53^ are possibly the surface expressions of active fluid flows associated with age-progressive arc magmatism^54^; however, the details remain unclear, which is geographically consistent with the slab edge at a depth of ca. 300 km at the subduction interface (Fig. 4a,b).

The deep southern Mariana Trench (< 18° N) provides additional topographic evidence to potentially infer the straight subduction in the southern Marianas contributing to the stress accumulation and formation of the deepest trench on Earth (maximum of 10,920 m in the Challenger Deep). In the northern Marianas, the trench is shallower than 8000 m on average, differing from the southern Marianas (Fig. 4a,b). The plate dip downdip of the Challenger Deep is small at a depth < 30 km (Moho depth) (Fig. 4b) along the southern Mariana strike and becomes steep subduction at the Moho depth (Fig. 4b) due to slab bending. Similar slab bending at various slab segments is observed in the central and northern Marianas and occurs at average depths of 60–80 km. Hence, the temperature states at deep and shallow interfaces differ significantly, and the resultant slab is likely hotter at depth than previously considered.

Our calculation indicates that the subduction thermal regime in the Marianas is cold in shallow portions (< 50 km) and similar to the southern Izu‒Bonin arc and northeast Japan. In such a cold system, especially in shallow megathrusts, high-pressure metamorphic rocks are difficult to find. Our results suggest that at depths of < 40 km, slab metamorphism does not occur easily (Fig. 4c). However, the plunging down of the slab starts at a depth of 50 km, which likely composes a barrier for cold slab interiors to be directly transported to depth via the slab-bending region. Increased compress/tension stress leads to slab weakening and fractures in the bending portions, and brittle–viscously damaged slabs show a tendency for detachment at elevated mantle temperatures^55^. Hence, the weakened slab surface due to bending is a candidate to interpret the formation of high P–T conditions and deep slab metamorphism at depths of > 50 km below the Marianas (Fig. 4c), as evidenced by the forearc metamorphosed basalts and serpentine exhumation^56^ and the high-temperature metamorphic rocks (ca. 1.6 GPa, 50 km, and 590 °C) of the blueschist clasts in the Mariana, which indicate that the thermal regime was warmer than typical oceanic subduction^57^. Combined with the U–Pb rutile and zircon geochronology, the P–T data suggest that the blueschist clasts record initially warm conditions during the early initiation of Pacific plate subduction^58–60^. During the early stages of subduction, conditions are generally warmer, as the plate subducts at a shallower angle, and the ‘dragging down’ of geotherms at the base of the overlying mantle wedge has not yet been significantly achieved^61^. This is in line with warm P–T estimates from newly initiated subduction zones that have also been recorded by high-pressure mafic rocks^60^. In addition, seismic tomography showed that the subducting Pacific plate below the Marianas is characterized by low attenuation at depths greater than 100 km, but high attenuation is found in the plate between depths of 50 and 100 km due to hydration and/or melting^62^. A moderate to high subduction temperature for the deep subducted plate and slab residuals at depths of > 50 km below the Marianas is supported by geological, geophysical, and geodynamic evidence.

### Comparison with previous thermal modeling results and exhumed rocks

**Figure 6:**
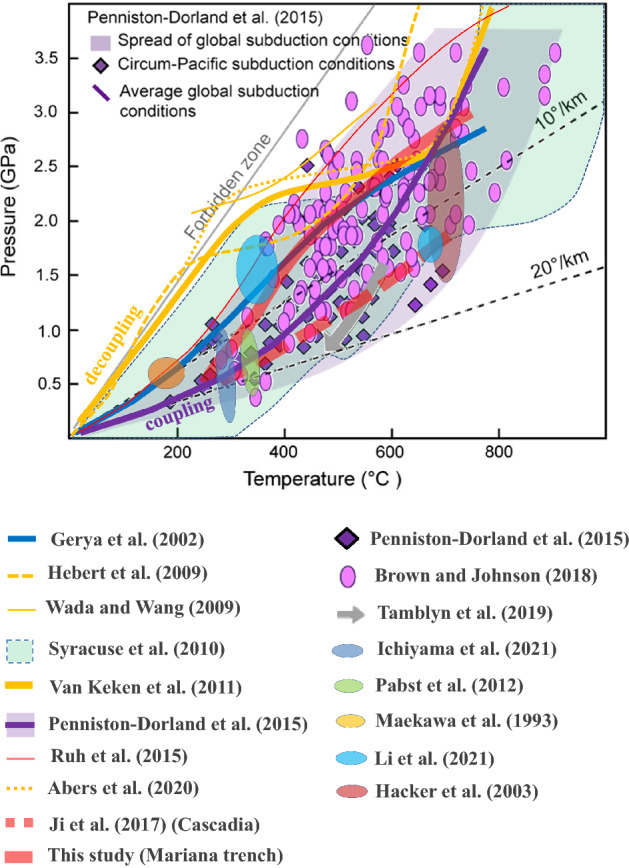
Comparison of the subduction P–T conditions between this study (thick red curve), previous thermal models (other colored curves) and the observations of exhumed rocks (colored ellipses, diamonds and arrows). Our temperature results (thick red curve) are higher than those of Wada and Wang^9^ (yellow thin line) and other cold-type model results^68–70^ by 200–300 °C but compare well with the P‒T conditions of observations of other thermal models^10,61^, exhumed rocks^63,64^, and other rock evidence (colored ellipses).

A comprehensive comparison between the thermal models and exhumed rock P–T conditions has been performed (Fig. 6). First, globally observed exhumed rocks provide a reference for P–T subduction conditions^63,64^. The other rock or clast samples include the studies from Maekawa et al.^65^, Hacker et al.^50^, Tamblyn et al.^57^, Li et al.^66^, Ichiyama et al.^67^ and so on. Among them, Tamblyn et al.^57^ suggested much warmer P–T conditions for the Marianas according to rock/clast records but still within the P–T range benchmarks from Penninston-Dorland et al.^63^ and Brown and Johnson^64^ (gray arrow in Fig. 6). All of these studies involving petrological evidence indicated warm subduction P–T conditions with an interface temperature of > 200 °C at a depth of 30 km, which is consistent at different depths with this study (cold megathrusts) and our previous study for Cascadia (warm megathrusts).

Second, considering the criteria of whether the interplate temperature exceeds 200 °C at a depth of 30 km (1 GPa, Moho depth), previous thermal models can be separated into two groups, i.e., cold-type (< 200 °C) models^9,17,68,70^ and decoupling models in Sycrause et al.^10^ and warm-type (> 200 °C) models^31,61,71^. Since cold-type models usually incorporate the slab–mantle decoupling layer into modeling and prescribe a maximum decoupling depth, the calculation is generally colder than the warm-type models by 100–300 °C at depths of 30–60 km (1–2 GPa). However, at a depth of 90 km (3 GPa), interplate temperatures of the two groups are calculated in a similar range (500–800 °C) at subvolcanic depths, with their upper limits constrained by the mantle melting solidus.

Third, compared with the globally exhumed rock records, Penninston-Dorland et al.^63^ found that the model results in van Keken et al.^17^ are colder by 200–300 °C than those shown by rock evidence. In contrast, our plate-coupling models for the Mariana (red thick curve) and the cold-type models, including Gerya et al.^61^, obtained a slab temperature that is hotter than that of the plate-decoupling warm-type models by ca. 200 °C, which is seemingly more consistent with the observed exhumed rocks, as suggested by Penninston-Dorland et al.^63^ and Brown and Johnson^64^.

In addition, we performed a detailed comparison between our result and that in Wada and Wang^51^, in which the cross-section temperature variation along the profile crossing Pagan volcano was presented (Fig. S2). They suggested that the slab depth beneath Pagan volcano is approximately 110 km, which is determined by global teleseismic earthquake relocation with improved travel times and procedures for depth determination^72,73^. However, we found that slab geometry data obtained from Slab2^20^ are slightly different below Pagan volcano (Fig. S2). In addition, the plate decoupling hypothesis leads to a forearc interplate temperature as low as < 400 °C and an abrupt increase from 400 to 1000 °C beneath the arc, but our results show a progressive temperature elevation on the upper slab surface.

### 3-D slab dehydration, thermal gradient, and subduction earthquakes

The seismic events detected on the Mariana megathrust are more clustered in the southern Marianas, especially in the subarc and forearc regions south of 15° N (Fig. 4d), than those north of 15° N and in the northern Marianas. This contrast may be controlled by slab dehydration embrittlement, as determined by the subduction angle and pore overpressurization^74,75^ and possibly by the subsequent fault strength changes due to slab morphologic variability, as well as fluid channelization in the forearc mantle corner^76,77^. In general, similar to the situations in other subduction zones, the oldest subducted oceanic seafloor produces interface earthquakes under the control of the hydrothermal state of the incoming plate. Amphibolitization, eclogitization, and possible harzburgitization fronts involve the majority of subduction earthquakes in the Marianas, including deep seismic events.

Slab dehydration > 0.02 wt%/km east of the Mariana Islands indicates slab dewatering and fluid release from the subduction interface (Fig. 4d), which is more conspicuous in width on the plate interface than the counterparts in the central and northern Mariana, partially due to a rapidly descending old and cold oceanic plate with a large dip angle beneath the southern Marianas. The interplate geotherms from 300 to 700 °C in the southern Marianas also feature a slower thermal transition along the megathrust compared with those in the central and northern Marianas (Fig. 4d) over a distance of approximately 200 km updip of the interface portion beneath Guam and adjacent islands, where interplate earthquakes are more frequently observed compared with the other portions at the interface beneath the Marianas (Fig. 4d). Therefore, slab dehydration is considered to be one of the important factors influencing the generation of interface earthquakes beneath the Marianas.

The frequent seismicity located in the southern Marianas (12–14° N) corresponds to a temperature gradient inside the slab higher than 10 °C/km, as shown by the temperature gradient result (Fig. S3). Zheng and Chen^78^ also suggested that the dehydration of amphibole generally occurs at a high thermal gradient of > 11 °C/km. The molybdenum isotopes in the Marianas indicate that the serpentinization of the forearc mantle may result from shallow slab dehydration at < 80 km and is further fluxed by fluid/melt at depths > 80 km, as influenced by the subducting slab dragging down the serpentinized forearc mantle^66^. The cold subduction plate of the Marianas mostly dehydrates and releases abundant fluids beneath the forearc at depths of 70–80 km^79^. Our model supports the idea that the outflux peaks at approximately 70 km due to slab dehydration of amphibolitization to eclogitization at the front (Fig. 4d) and the largest interplate temperature gradient at this depth (Fig. S3). The magnitudes of earthquakes occurring at depth are influenced by the presence of a high temperature gradient and high pore fluid pressures. Released fluids lead to pore fluid pressure increases and facilitate the occurrence of intermediate–small earthquakes^80^.

### Slab dehydration and water flux estimate

The increases in the plate deflection and flexural bending from the northern to southern Marianas and the water flux in the south have been calculated to be 15% greater than those in the northern and central Marianas^48^, which is consistent with our result that slab dehydration and temperature gradients are enhanced in the southern Marianas due to straight subduction and megathrust segmentation and heterogeneity (Figs. 4d and S3).

Shallow slab dewatering is evidenced by fluid expulsion in accretionary prisms and active serpentinite seamounts in the Mariana forearc^81^. Most variations in composition among primitive basalts from the Mariana Trough can be explained by melting mixtures of ultramafic mantle sources and water-rich components^82^. At Anatahan–Pagan (16–18° N), the seismic images of the Mariana Trench show that the low-velocity zone within the subducted slab mantle at a depth of approximately 40 km preserves the original thickness but exhibits a smaller reduction in velocity (approximately 4.1 km s^−1^); thus, the additional velocity reduction (approximately 0.3 km s^−1^) with respect to the surrounding upper mantle can be attributed to pore water in cracks^18^. The subducted slab at depths from 40 to 60 km has a reduced S-wave velocity due to slab serpentinization from the interface where the fluids obtained probably originate from deeper slab dehydration. The dehydration depths (40–60 km) are consistent with our dehydration distribution at Anatahan–Pagan (Fig. 4d). Cai et al.^18^ further interpret seismic images as strong evidence for a (24 ± 5)-km-thick, partially serpentinized (2 wt% water) slab mantle layer and estimate the water flux for the Marianas to be 79 ± 17 TgMyr^−1^ m^−1^, which is 4.3 ± 0.8 times larger than a previous estimate^16,17^ that assumed a 2-km-thick, partially serpentinized slab mantle (2 wt% water). Our estimate of the water flux for the Marianas is based on the thickness of the slab mantle layer (30–100 km), its partial serpentinization (2 wt% water or more), and the subduction velocity varying along strike (2–3.5 cm/yr) of approximately 50–300 TgMyr^−1^ m^−1 (^the weight of water input after each meter of subduction and each million-year period); this estimate is largely dependent on the plate geometry and has a magnitude similar to that of the water flux estimate that was obtained^18^ at Anatahan–Pagan (16–18° N), which has a cold mantle wedge within the Mariana convergence zone.

## Conclusions

Through 3-D thermomechanical modeling of the Mariana subduction megathrust, we draw the following conclusions:

1. The concave slab geometry associated with the complex plate tectonics in the Marianas features a thermal regime with a heterogeneous interface that changes along strike and the formation of a cold mantle wedge in the central Marianas.
2. The large dip and straight subduction of the Pacific plate in the southern Marianas are associated with a large temperature gradient and strong slab dehydration. Amphibolitization, eclogitization, and harzburgitization influence the occurrence of interface and intraslab earthquakes below the Marianas.
3. High slab dehydration (> 0.02 wt%/km) east of the Mariana Islands indicates enhanced fluid release and potential migration along the megathrusts that are comparable to the water flux estimate.

## Supplementary Information

Supplementary Information.

## Data Availability

Data are available in the tables and figures of this study or from the authors upon request.
