# Supplementary material for: Subduction thermal regime, petrological metamorphism and seismicity under the Mariana arc
Source: Sci Rep. 2023 Feb 2;13:1948. doi: 10.1038/s41598-023-29004-1 (PMC9894833; doi:10.1038/s41598-023-29004-1)
Supplement: Supplementary file 1 — Supplementary Information. [file 41598_2023_29004_MOESM1_ESM.docx]

**Supporting Information for**

Subduction thermal regime, petrological metamorphism and seismicity under the Mariana arc

Rui Qu^1, 2^, Weiling Zhu^1, 2^, Yingfeng Ji^1, 2^, Chaodi Xie^3^, Deng Zeng^1, 2^, Fan Zhang^4, 5^

1. *State Key Laboratory of Tibetan Plateau Earth System, Environment and Resources (TPESER), Institute of Tibetan Plateau Research, Chinese Academy of Sciences, Beijing 100101, China*
2. *University of Chinese Academy of Sciences, Beijing 100049, China*
3. *Department of Geophysics, School of Earth Science, Yunnan University, Kunming 650091, China*
4. *Key Laboratory of Ocean and Marginal Sea Geology, South China Sea Institute of Oceanology, Innovation Academy of South China Sea Ecology and Environmental Engineering, Chinese Academy of Sciences, Guangzhou 510301, China*
5. *Southern Marine Science and Engineering Guangdong Laboratory, Guangzhou 511458, China*

**Contents of this file**

1. Methods and model setting

2. Tables S1-S2

3. Figures S1-S4

4. References

1. **Methods and model setting**

Based on the thermomechanical model Stag3D (Tackley and Xie, 2003) and the finite difference method (FDM), we investigate the in situ along-strike slab thermal variation in megathrusts and the petrological metamorphic changes in the incoming oceanic lithosphere. An anelastic liquid approximation and the equations of conservation of mass, momentum, and energy are used in this study:

$\nabla\cdot\left\{\rho_{s}(z,T_{s})\mathbf{v}\right\}=0$, (1)

$-\frac{\partial P}{\partial x_{i}}+\frac{\partial\tau_{ij}}{\partial x_{j}}-\delta_{i3}\rho_{s}g\alpha_{0}(T-T_{s})=0$, (2)

$\rho c_{p}(\frac{\partial\text{T}}{\partial t}+\mathbf{v}\cdot\nabla T)=k\nabla^{2}T+\eta(\nabla v)^{2}+\rho g\alpha Tv_{z}+\rho H_{r}$, (3)

where $P$ is the pressure deviation from hydrostatic pressure, $\alpha_{0}$ is the reference thermal expansivity, $\tau_{ij}$ ($i$,$j$= 1, 2, 3) is the stress tensor and $\delta_{ij}$ is the Kronecker delta. The energy equation includes the advection term, thermal diffusion term, viscous dissipation term, adiabatic heating term, and radioactive heating term. The main model parameters are tabulated in Tables S1 and S2.

The viscous flow law for wet olivine (Burkett and Billen, 2010) following laboratory experiments is included in this study. The deformation of olivine occurs by both diffusion creep ($df$) and dislocation creep ($ds$), where each mechanism accommodates a portion of the total strain rate (Hirth and Kohlstedt, 2003):

$\dot{\varepsilon}_{t}=\dot{\varepsilon}_{df}+\dot{\varepsilon}_{ds}$. (4)

The composite upper mantle viscosity under a given condition is

$\eta_{comp}=\frac{\eta_{df}\eta_{ds}}{\eta_{df}+\eta_{ds}}$, (5)

where ηdf and ηds are the diffusion creep and dislocation creep viscosities for olivine, respectively. The general viscosity law is

$\eta_{df,ds}=\left( \frac{d^{p}}{A_{0}C_{OH}^{r}} \right)^{\frac{1}{n}}\dot{\varepsilon}_{E}^{\frac{1-n}{n}}\exp\left( \frac{E_{0}+P_{l}V_{0}}{n_{0}RT_{a}} \right)$. (6)

Here, $\dot{\varepsilon}_{E}=(\frac{1}{2}\dot{\varepsilon}_{ij}\dot{\varepsilon}_{ij})^{\frac{1}{2}}$ is the square root of the second invariant of the strain rate tensor (e.g., Ranalli, 1995), Ta is the absolute temperature, R is the gas constant, and Pl is the lithostatic pressure. The values of the model parameters for the diffusion and dislocation creep of olivine are tabulated in Table S1. The trenchward thermal structure follows the Global Depth and Heat (GDH1) model (Stein and Stein, 1992; Grose and Afonso, 2013):

$T(z,t_{oc})=T_{m}\left[ \frac{z}{d_{0}}+\frac{2}{\pi}\sum_{n-1}^{\infty} \frac{1}{n}sin(\frac{n\pi z}{d_{0}})exp(\frac{-n^{2}\pi^{2}{\kappa t}_{oc}}{{d_{0}}^{2}}) \right]$ (7)

The time-dependent thermal boundary condition includes the initialization time. $T(z,t_{oc})$ is the temperature at depth z and plate age toc along the trench, Tm is the lithospheric basal temperature, $\kappa$ is the thermal diffusivity, and d0 is the adiabatic heating layer minimum depth.

The model involves 3-D geometric data for the incoming plate updated through seismic tomography (Slab2, Hayes et al., 2018) and real subduction velocities from the MORVEL (Argus et al., 2011; DeMets et al., 2010) data sets. The age of the oceanic plate follows the oceanic seafloor age according to EarthByte (Müller et al., 2008) estimated at the trenchward model boundary. The thickness of the incoming plate is estimated according to the plate age (Yoshii, 1975). The incoming oceanic plate is composed of a MORB layer at the top with a thickness of 7 km underlain by an ultramafic rock layer (Hacker et al., 2003). The model dimensions are 1350 km×750 km×400 km (along-arc length×across-arc length×depth) and 80×80×100 grids. The temperature boundary condition agrees with the plate cooling model (Grose and Afonoso, 2013). The bottom of the slab and the perpendicular plane are prescribed as adiabatic and permeable, and the top surface is set to be a fixed temperature (0°C) and rigid. The subduction velocities inside a prescribed 3-D constrained volume of the oceanic lithosphere are given based on the kinematic plate subduction modeling method (Ji et al., 2016; Ji et al., 2017c):

, (8)

, (9)

, (10)

while

$a(x,y)=\frac{1}{2}\{Z(x+\Delta x,y)-Z(x-\Delta x,y)\}\cdot\frac{z_{\max}}{x_{\max}}$, (11)

$b(x,y)=\frac{1}{2}\{Z(x+\Delta x,y+\Delta y)-Z(x+\Delta x,y)+Z(x-\Delta x,y)-Z(x-\Delta x,y-\Delta y)\}\cdot\frac{z_{\max}}{y_{\max}}$.

(12)

Here, v is the subduction velocity, and Δ is the interval between two neighboring nodes along the axes. $x_{\max}$, $y_{\max}$, and $z_{\max}$ indicate the model lengths along the x, y, and z axes, respectively.

Observations of surface heat flow from the Global Heat Flow Database (Pollack et al., 1993) and heat flow values from Curie point depth estimates (Li et al., 2017) are used to constrain the model (Fig. S1). Our model follows the trench temperature boundary of the plate cooling model (Stein and Stein, 1992; Grose and Afonso, 2013). We prescribe the subduction time to be at least 20 Myr to ensure that the model reaches a steady thermal state with a temperature variation < 10℃ over time with a lapse time of $\geq$ 5 Myr. We tested the resolution and found that the temperature variance was < 1% with a maximum temperature variance < 1.9% between meshes of 80 × 80 × 100 and 96 × 96 × 100. We performed sensitivity tests to investigate the robustness of our modeling results and varied the mantle viscosity from 0.5×10^20^ Pa s to 1.5×10^20^ Pa s and the mantle density from 3250 kg/m^3^ to 3350 kg/m^3^. We present the benchmark model results as deviations from the reference models (ΔT and ΔH_2_O) and show these results at different depth levels within the oceanic slab. The tests show that mantle density variations (±50 kg/m^3^) and mantle viscosity variations (±0.5×10^20^ Pa s) induce small temperature variations of <10°C at depth inside the slab. The maximum mantle viscosity at a depth of < 400 km is prescribed to be 7.37×10^20^ (10^20.87^) Pa according to the VM2 mantle viscosity model (e.g., Peltier, 2011). Considering that the values of the parameters are yet controversial, it better to constrain thermal models in priority based on the observation of the surface heat flow (Fig. S1) and further if possible including the results of seismic inversion.

Ultramafic mantle rocks such as harzburgite (olivine + orthopyroxene) represent the dominant rock type in mantle wedges and the uppermost oceanic mantle, and depleted lherzolite (olivine+orthopyroxene+clinopyroxene) is considered subordinate (Hacker et al., 2003). Seismological studies support the hypothesis that harzburgite represents the principal rock type in the upper mantle. The observed P wave speeds from White et al. (1992) for the oceanic lower crust and mantle compared with P wave speeds for various rocks at 200 MPa (Hacker et al., 2003) indicate that most oceanic uppermost mantle (suboceanic mantle) velocity measurements are best explained in terms of spinel harzburgite mantle composition. Due to the above reasons, in our petrological modeling approach, harzburgite is assumed to be the dominant ultramafic rock.

We established a P–T-wt%-facies database according to Omori et al. (2009) (MORB) and Hacker et al. (2003) with a P–T grid interval of 0.04 GPa (1.2 km) and 5°C. The temperature and pressure at every P–T grid point are calculated from our 3D thermal model. The pressure (GPa) at every grid point is obtained by converting its depths (km) through preliminary reference earth model (PREM) parameters. Using the temperature and pressure provided by the numerical simulation, we estimate both each facies domain and the corresponding water content (wt%) at every grid. Through the interpolation method, we obtain the intraslab water content distribution (wt%) at various depths.

To calculate the inner-slab slab dehydration, the result of the 3-D water content at every grid is utilized. Slab dehydration (wt%/km) indicates the change in rock saturation water content (wt%) via a distance (km) in the subduction direction between neighboring grids. We prescribe the slab to be divided into >70 layers according to the mesh number, with the layer surface parallel to the plate interface. Then, the water content at a point on a layer surface is calculated from the water content at the grids surrounding this point using an interpolation method. Thus, the water content at each point on every layer surface is obtained. Next, the water content value difference between two neighboring points in the subduction direction is divided by the point distance (which can be derived using the horizontal distance and vertical distance), and then slab dehydration at each point on a layer can be obtained. Based on these steps, the intraslab dehydration distribution is well calculated. Slab minerals vary in saturation at each subduction stage. Thus, slab dehydration (wt%/km) reflects the efficiency of fluid production by crystalline breakdown and fluid relaxation during subduction along the slab geometry.

Surface heat flow and Curie depth are correlated, following a theoretical thermal conduction relationship (e.g., Li et al., 2011, 2013):

$Q_{s}=K\frac{T_{c}-T_{0}}{Z_{b}-Z_{s}}+h_{r}^{2}H_{0}\frac{e^{\frac{{-Z}_{b}}{h_{r}}}-e^{\frac{-Z_{s}}{h_{r}}}}{Z_{b}-Z_{s}}+h_{r}H_{0}e^{\frac{{-Z}_{s}}{h_{r}}}$ (13)

where Q_s_ is the surface heat flow, T_c_ is the Curie temperature at the Curie depth Z_b_, T_0_ is the temperature at the surface elevation Z_s_, K is the average thermal conductivity of the magnetic layer (Table S2), H_0_ is the heat production rate at the surface, and h_r_ is the characteristic drop-off of heat production. The equation shows a nonlinear inverse relationship between heat flow and Z_b_. For the oceanic lithosphere, we assume H_0_ = 1.37 μW/m^3^, h_r_ = 5.0 km, T_c_ = 550 °C, T_0_ = 5 °C, and Z_s_ = 4 km. For continents, we take H_0_ = 2.0 μW/m^3^, h_r_ = 10.0 km, and Z_s_ = -1 km to account for the larger radioactive contribution (Turcotte and Schubert, 2002). High heat flow measurements tend to correlate with small Curie depths, and vice versa. The continental data can be best fitted with the theoretical curve of an average thermal conductivity K of ~2.5 W/m°C, and most oceanic data are best fitted with an average K = ~2.0 W/m°C (Li et al., 2013). These conductivities are compatible with those of granite and basalt (e.g., Turcotte and Schubert, 2002). The synthetic modeling suggests that the largest error in the estimated Curie depths using the linearized centroid method will not reach 35%, and the uncertainty of the surface heat flow is expected to be <20 mW/m^2^ due to the selected fractal exponent and wavenumber bands for linear regressions and observed surface heat flow in plate convergence zones (e.g., Li et al., 2013, 2017).

The model settings combine effective simulation methods used in active convergence zones, such as the reconstruction of oceanic-continental cold subduction in eastern Japan (Ji et al., 2017b, 2017c), Hikurangi (Suenaga et al., 2018), and Sumatra (Ji et al., 2021) and warm subduction in southwestern Japan (Ji et al., 2016; Ji and Yoshioka, 2017), Ryukyu (Suenaga et al., 2021), and Cascadia (Ji et al., 2017a). These studies on 3-D thermomechanical models are focused on subducted plates with topographically changing dip angles with comparison with the distribution of subduction zone earthquakes (e.g., Fig. S4 for this study).

**Table S1. Main model parameters**

| Model Parameters Value Units |
| --- |
| Standard density 3300^a^ kg·m^-3^ |
| Standard [thermal expansion](dict://key.0895DFE8DB67F9409DB285590D870EDD/coefficient%20of%20thermal%20expansion) 3×10^-5b^ K^-1^ |
| Standard temperature 1600 K |
| Standard thermal conductivity 2.9^c^ W·m^-1^·K^-1^ |
| Radioactive heat generation rate in the mantle 2.245×10^-13a^ W·m^-3^ |
| Standard specific heat at constant pressure 1200^a^ J·kg^-1^·K^-1^ |
| Standard thermal diffusivity 7.6×10^-7d^ m^2^·s^-1^ |
| Standard [viscosity](dict://key.0895DFE8DB67F9409DB285590D870EDD/coefficient%20of%20viscosity) 1×10^20c^ Pa·s |
| $v$Subduction velocity 6.8–8.0^e^ cm·y^-1^ |

|  |  | Diffusion  creep^f^ | Dislocation creep^f^ |
| --- | --- | --- | --- |
| $n_{0}$ | Stress exponent | 1.0 | 3.5 |
| $A_{0}$ | Preexponential factor | 1.0 | 9.0×10^-20^ |
| $C_{OH}$ | OH concentration (H/10^6^ Si) | 1000 | 1000 |
| $r$ | C_OH_ exponent | 1.0 | 1.2 |
| $E_{0}$ | Activation energy (kJ/mol) | 335 | 480 |
| $V_{0}$ | Activation volume (m^3^/mol) |  |  |
|  | Upper mantle | 4.0×10^--6^ | 11.0×10^--6^ |
|  | Lower mantle | 1.5×10^--6^ | - |
| $d$ | Grain size (μm) |  |  |
|  | Upper mantle | 10,000 | - |
|  | Lower mantle | 40,000 | - |

^a^ Wang et al. (1995) ^b^ Iwamori (1997) ^c^ Christensen (1996) ^d^ Yoshioka and Murakami (2007)

^e^ Argus et al. (2011) ^f^ Hirth and Kohlstedt (2003)

**Table S2 Parameters for model domains**

| Domains | Upper crust | Lower crust | Slab | Mantle | Accretionary prism |
| --- | --- | --- | --- | --- | --- |
| Density (kg/m^3^) | 2600 | 2900 | 3300 | 3300 | 2600 |
| Viscosity (Pa·s) | 1×10^20^ | 1×10^20^ | 1×10^20^ | 1×10^20^ | 1×10^20^ |
| Radioactive heat (W/m^3^) | 7.3e-10 | 1.4e-10 | 2.245^e-13^ | 2.245^e-13^ | 7.3e-10 |
| for magnetic layers:  oceanic  continental | 1.37e-6  2.0e-6 |  |  |  |  |
| Thermal conductivity (W/m·K) | 2.5 | 2.5 | 2.5 | 2.5 | 1.4 |
| for magnetic layers:  oceanic  continental | 2.0  2.5 |  |  |  |  |


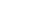


Fig. S1 (a) Spatial distribution of the surface heat flow calculated in this study. The observed and calculated surface heat flows are compared along trench-normal profiles “b” to “i” in panels (b) to (i). (b) Observed and calculated heat flows along profile b (y = 202.5 km) in (a). Purple diamonds and blue squares denote heat flow data from the global heat flow database (Pollack et al., 1993) and heat flow from Curie point depth estimates (Li et al., 2017), respectively, within a width of 25.3 km along the profile. The red curve indicates the calculated heat flow along the profile. (c)-(i) Along profiles c-i. The thermal conductivities K are assumed to be 1.5 W/m°C for the ocean (Grevemeyer et al., 2003; Li et al., 2017) and 2.5 W/m°C for the continent (Li et al., 2017). The figure was created by using the Generic Mapping Tools (GMT) (Wessel and Smith, 1998) (version: GMT 4.5.7, URL link: https://www.generic-mapping-tools.org/download/).

Fig. S2


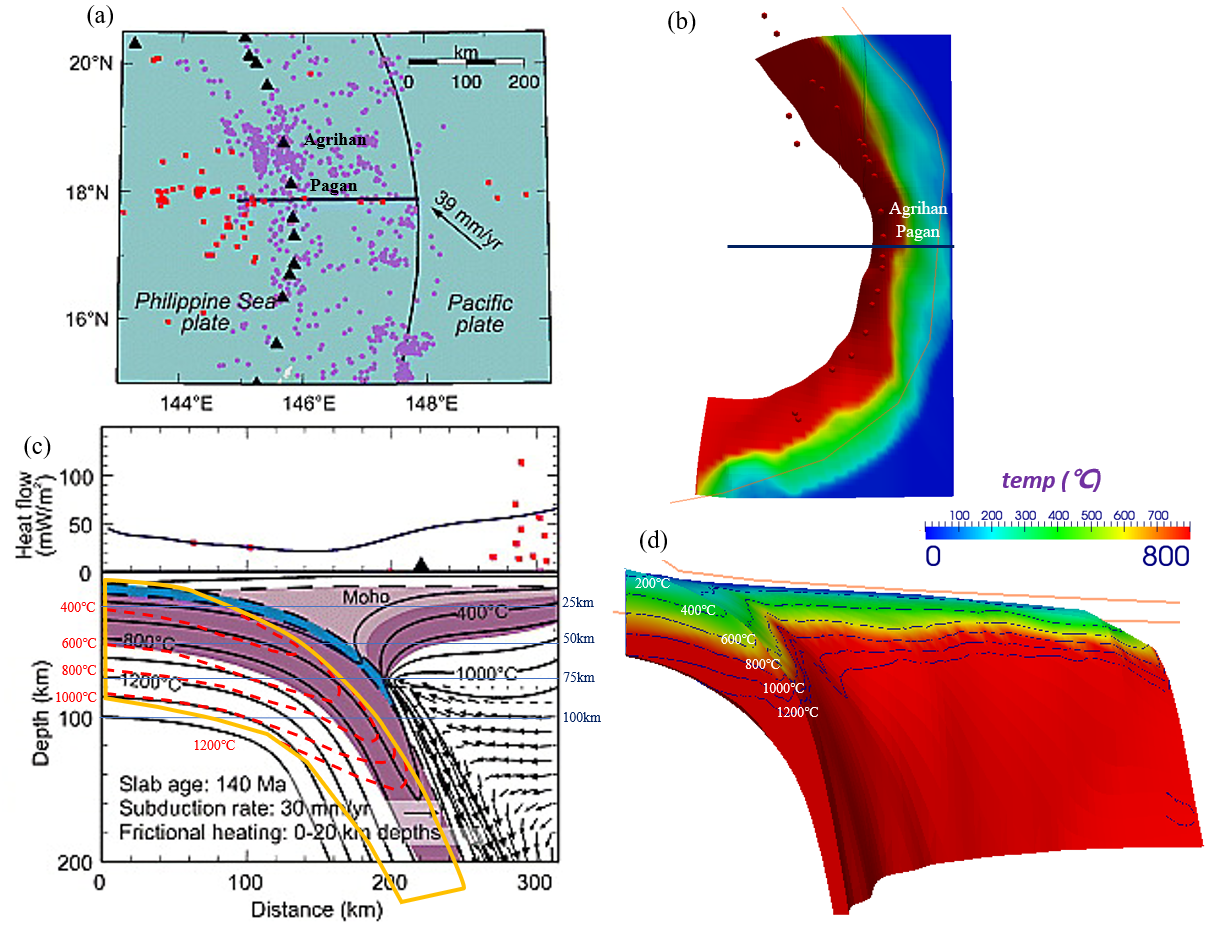


Fig. S2 Comparison of model results between this study and Wada and Wang (2009). (a) The profile was used to estimate the cross-section thermal structure in Mariana by the 2-D model (Wada and Wang, 2009). (b) Obtained the same profile from the 3-D model in this study. (c) Slab geometry difference and calculated thermal result difference between Wada and Wang (2009) and this study. Black curves and symbols are from Wada and Wang (2009), while yellow curves (slab surface) and red curves (intraslab thermal contours) are from this study. (d) Cross-section thermal structure result along the same profile in this study from a 3-D perspective. Figures were created by using the Generic Mapping Tools (GMT) (Wessel and Smith, 1998) (version: GMT 4.5.7, URL link: <https://www.generic-mapping-tools.org/download/)> and Paraview (version: Paraview 5.4.1, URL link: https://www.paraview.org/download/).

Fig. S3

Fig. S3 Slab thermal gradient in the subduction direction along the surfaces. Colored spheres indicate the interplate earthquakes and intraslab earthquakes, which occurred during the period from Jan. 1, 2000, to Dec. 31, 2009 (IRIS). The figure was created by using the software Paraview (version: Paraview 5.4.1, URL link: https://www.paraview.org/download/).

Fig. S4

Fig. S4 Seismic activity map of the Marianas (including the intraplate events occurring in the overriding plate). Background colors indicate the surface topography (ETOPO; Smith and Sandwell, 1997). Red triangles indicate active volcanoes (Siebert et al., 2010). Red curve marks convergent plate boundary (Bird, 2003). Colored spheres indicate regular earthquakes that occurred during the period from January 1, 2001, to December 31, 2009 (IRIS; Trabant et al., 2012). Yellow arrows indicate the motion of the Pacific Plate toward the Philippine Sea Plate (MORVEL; DeMets et al., 1990, 2010; DeMets and Dixon, 1999). (a) the hypocenter depth range is 0-130 km. (b) the hypocenter depth range is 130-650 km. The figure was created by using the Generic Mapping Tools (GMT) (Wessel and Smith, 1998) (version: GMT 4.5.7, URL link: https://www.generic-mapping-tools.org/download/).

**References**

Abers, G.A., van Keken, P.E., Wilson, C.R. (2020). Deep decoupling in subduction zones: Observations and temperature limits. Geosphere 16, 1408-1424.

Agard, P., Yamato, P., Soret, M., Prigent, C., Guillot, S., Plunder, A., B. Dubacq, A. Chauvet, Monié, P. (2016). Plate interface rheological switches during subduction infancy: Control on slab penetration and metamorphic sole formation. Earth Planet. Sci. Lett., 451, 208-220

Anderson, M., Chadwick Jr., W., Hannington, M., Merle, S., Resing, J., Baker, E., Butterfield, D., Walker, S., N. Augustin. (2017). Geological interpretation of volcanism and segmentation of the Mariana back-arc spreading center between 12.7°N and 18.3°N, Geochem. Geophys. Geosyst., 18, 2240–2274.

Argus, D.F., Gordon, R.G., DeMets, C. (2011). Geologically current motion of 56 plates relative to the no-net-rotation reference frame. Geochemistry, Geophysics, Geosystems, 12(11).

Ashi, J., Tokuyama, H., Taira, A. (2002). Distribution of methane hydrate BSRs and its implication for the prism growth in the Nankai Trough. Marine Geology 187, 177-191.

Ashi, J., Tokuyama, H., Ujiie, Y., Taira, A. (1999). Heat flow estimation from gas hydrate BSRs in the Nankai Trough: Implications for thermal structures of the Shikoku Basin. Eos Trans. AGU 80.

Becker, T.W., Faccenna, C. (2009). A review of the role of subduction dynamics for regional and global plate motions. In Subduction zone geodynamics (pp. 3-34). Springer, Berlin, Heidelberg.

Billen, M. I. (2008), Modeling the dynamics of subducting slabs, Annu. Rev. Earth Planet. Sci., 36, 325– 356.

Bird, P. (2003). An updated digital model of plate boundaries. Geochemistry, Geophysics, Geosystems, 4(3).

Boulegue, J., Benedetti, E. L., Dron, D., Mariotti, A., Letolle, R. (1987). Geochemical and biogeochemical observations on the biological communities associated with fluid venting in Nankai Trough and Japan Trench subduction zones. Earth and planetary science letters, 83(1-4), 343-355.

Burkett, E.R., Billen, M.I. (2010). Three-dimensionality of slab detachment due to ridge-trench collision: Laterally simultaneous boudinage versus tear propagation, Geochem. Geophys. Geosyst., 11, Q11012, doi:[10.1029/2010GC003286](http://dx.doi.org/10.1029/2010GC003286).

Brown, M., Johnson, T. (2018). Secular change in metamorphism and the onset of global plate tectonics. American Mineralogist 103, 181-196.

Cai, C., Wiens, D. A., Shen, W., Eimer, M. (2018). Water input into the Mariana subduction zone estimated from ocean-bottom seismic data. Nature, 563(7731), 389-392.

Condit, C.B., Guevara, V.E., Delph, J.R., French, M. E. (2020). Slab dehydration in warm subduction zones at depths of episodic slip and tremor. Earth and Planetary Science Letters, 552, 116601.

DeMets, C., Gordon, R.G., Argus, D.F. (2010). Geologically current plate motions, Geophys. J. Int., 181, 1, 1-80, doi: 10.1111/j.1365-246X.2009.04491.x.

DeMets, C., Dixon, T.H. (1999). New kinematic models for Pacific‐North America motion from 3 Ma to present, I: Evidence for steady motion and biases in the NUVEL‐1A model. Geophysical Research Letters, 26(13), 1921-1924.

Emry, E.L., Wiens, D.A., Shiobara, H., Sugioka, H. (2011). Seismogenic characteristics of the Northern Mariana shallow thrust zone from local array data. Geochemistry, Geophysics, Geosystems 12.

Engdahl, E.R., van der Hilst, R., Buland, R. (1998). Global teleseismic earthquake relocation with improved travel times and procedures for depth determination. Bulletin of the Seismological Society of America 88, 722-743.

England, P., Engdahl, R., Thatcher, W. (2004). Systematic variation in the depths of slabs beneath arc volcanoes. Geophysical Journal International 156, 377-408.

Faccenna, C., Holt, A.F., Becker, T.W., Lallemand, S., Royden, L.H. (2018). Dynamics of the Ryukyu/Izu-Bonin-Marianas double subduction system. Tectonophysics, 746, 229-238.

Fryer P, Wheat CG, Mottl MJ. 1999. Mariana Blueschist mud volcanism: implications for conditions within the subduction zone. Geology 27, 103–106.

Fryer, P., Wheat, C. G., Williams, T., Kelley, C., Johnson, K., Ryan, J., ... Pomponi, S. (2020). Mariana serpentinite mud volcanism exhumes subducted seamount materials: implications for the origin of life. Philosophical Transactions of the Royal Society A, 378(2165), 20180425.

Gerya, T. V. (2011). Intraoceanic subduction zones. In Arc-continent collision (pp. 23-51). Springer, Berlin, Heidelberg.

Gerya, T. V., Bercovici, D., Becker, T. W. (2021). Dynamic slab segmentation due to brittle–ductile damage in the outer rise. Nature, 599(7884), 245-250.

Gerya, T.V., Stöckhert, B., Perchuk, A.L. (2002). Exhumation of high‐pressure metamorphic rocks in a subduction channel: A numerical simulation. Tectonics 21, 6-1-6-19.

Grevemeyer, I., Diaz-Naveas, J.L., Ranero, C.R., Villinger, H.W., [Ocean Drilling Program Leg 202 Scientific Party](https://www.sciencedirect.com/science/article/pii/S0012821X03003030#!), (2003). Heat flow over the descending Nazca plate in central Chile, 32 S to 41 S: observations from ODP Leg 202 and the occurrence of natural gas hydrates. Earth Planet. Sci. Lett. 213 (3), 285–298.

Grose, C.J. Afonso, J.C. (2013). Comprehensive plate models for the thermal evolution of oceanic lithosphere, Geochem. Geophys. Geosyst., 14, 3751–3778, doi:[10.1002/ggge.20232](http://dx.doi.org/10.1002/ggge.20232).

Gvirtzman, Z., Stern, R.J. (2004). Bathymetry of Mariana trench‐arc system and formation of the Challenger Deep as a consequence of weak plate coupling. Tectonics, 23(2).

Hacker, B.R., Abers, G.A., Peacock, S.M. (2003). Subduction factory 1. Theoretical mineralogy, densities, seismic wave speeds, and H2O contents. J. Geophys. Res., 108, 2029, doi:10.1029/2001JB001127.

Hayes, G.P., Moore, G.L., Portner, D.E., Hearne, M., Flamme, H., Furtney, M., Smoczyk, G.M. (2018). Slab2, a comprehensive subduction zone geometry model. Science, 362(6410), 58-61

Hebert, L. B., Antoshechkina, P., Asimow, P., Gurnis, M. (2009). Emergence of a low-viscosity channel in subduction zones through the coupling of mantle flow and thermodynamics. Earth and Planetary Science Letters, 278(3-4), 243-256.

Hirth, G., Kohlstedt, D. (2003), Rheology of the upper mantle and the mantle wedge: A view from the experimentalists, in Inside the Subduction Factory, Geophys. Monogr. Ser., vol. 138, edited by J. Eiler, pp. 83 – 105, AGU, Washington.

Homrighausen, S., Hoernle, K., Zhou, H., Geldmacher, J., Wartho, J.A., Hauff, F., Werner, R., Jung, S., Morgan, J.P. (2020). Paired EMI-HIMU hotspots in the South Atlantic—Starting plume heads trigger compositionally distinct secondary plumes? Science advances 6, eaba0282.

Hyndman, R.D. (2013). Downdip landward limit of Mariana great earthquake rupture. J. Geophys. Res., 118, 5530–5549, <http://dx.doi.org/10.1002/jgrb.50390>.

Hyndman, R.D., McCrory, P.A., Wech, A., Kao, H., Ague, J. (2015). Mariana subductingplate fluids channelled to fore-arc mantle corner: ETS and silica deposition. J. Geophys. Res., 120, 4344–4358, <http://dx.doi.org/10.1002/2015JB011920.2015>.

Ichiyama, Y., Tsujimori, T., Fryer, P., Michibayashi, K., Tamura, A., Morishita, T. (2021). Temporal and spatial mineralogical changes in clasts from Mariana serpentinite mud volcanoes: Cooling of the hot forearc-mantle at subduction initiation. Lithos, 384, 105941.

Ishizuka, O., Tani, K., Reagan, M. K., Kanayama, K., Umino, S., Harigane, Y., Sakamoto, I., Miyajima, Y., Yuasa, M., Dunkley, D. J. (2011). The timescales of subduction initiation and subsequent evolution of an oceanic island arc. Earth Planet. Sci. Lett. 306, 229–240. doi:10.1016/j.epsl.2011.04.006.

Ishizuka, O., Hickey-Vargas, R., Arculus, R. J., Yogodzinski, G. M., Savov, I. P., Kusano, Y., McCarthy, A., Brandl, P. A., Sudo, M. (2018). Age of Izu-BoninMariana arc basement, Earth Planet. Sci. Lett. 481, 80–90. https://doi.org/10.1016/j.epsl.2017.10.023.

Ji, Y., Yan, R., Zeng, D., Xie, C., Zhu, W., Qu, R., Yoshioka, S., (2021). Slab dehydration in Sumatra: Implications for fast and slow earthquakes and arc magmatism. Geophys. Res. Lett., 48, e2020GL090576.

Ji, Y., Yoshioka, S. (2017). Slab dehydration and earthquake distribution beneath southwestern and central Japan based on three-dimensional thermal modeling, Geophys. Res. Lett., 44, 2679–2686, doi:[10.1002/2016GL072295](http://dx.doi.org/10.1002/2016GL072295)

Ji, Y., Yoshioka, S., Banay, Y.A. (2017a). [Thermal state, slab metamorphism and interface seismicity in the Cascadia subduction zone based on 3-D modeling: 3-D thermomechanical model for Cascadia](https://www.researchgate.net/publication/319574826_Thermal_state_slab_metamorphism_and_interface_seismicity_in_the_Cascadia_subduction_zone_based_on_3-D_modeling_3-D_thermomechanical_model_for_Cascadia?_iepl%5BviewId%5D=3yLR53ej2nHM1kNScBa5AsFd&_iepl%5BprofilePublicationItemVariant%5D=default&_iepl%5Bcontexts%5D%5B0%5D=prfpi&_iepl%5BtargetEntityId%5D=PB%3A319574826&_iepl%5BinteractionType%5D=publicationTitle), Geophys. Res. Lett., 44, 9242–9252, doi:10.1002/2017GL074826

Ji, Y., Yoshioka, S., [Manea](http://www.sciencedirect.com/science/article/pii/S0040195111003957), V., [Manea](http://www.sciencedirect.com/science/article/pii/S0040195111003957), M. (2017b). Seismogenesis of dual subduction beneath Kanto, central Japan controlled by fluid release, Scientific Reports, 7(1), 16864, doi:10.1038/s41598-017-16818-z

Ji, Y., Yoshioka, S., Manea, V. C., Manea, M., Matsumoto, T. (2017c). Three-dimensional numerical modeling of thermal regime and slab dehydration beneath Kanto and Tohoku, Japan, J. Geophys. Res. solid earth, 122, 332-353, doi:[10.1002/2016JB013230](http://dx.doi.org/10.1002/2016JB013230)

Ji, Y., Yoshioka S., Manea, V., Manea, M., Suenaga, N. (2019).　Three-Dimensional Thermal Structure, Metamorphism and Seismicity beneath north-central Chile, J. Geodynamics, 129, 299-312, https://doi.org/10.1016/j.jog.2018.09.004

Ji, Y., Yoshioka, S., Matsumoto, T. (2016). Three-dimensional numerical modeling of temperature and mantle flow fields associated with subduction of the Philippine Sea Plate, southwest japan. Journal of Geophysical Research: Solid Earth, 121(6), 4458-4482. doi:10.1002/2016JB012912.

Johnson LE, Fryer P. 1990. The first evidence for MORB-like lavas from the outer Mariana forearc; geochemistry, petrology, and tectonic implications. Earth Planet. Sci. Lett. 100, 304–316.

Kong, X., Li, S., Wang, Y., Suo, Y., Dai, L., Geli, L., Zhang, Y., Guo, L., Wang, P. (2018). Causes of earthquake spatial distribution beneath the Izu-Bonin-Mariana Arc. Journal of Asian Earth Sciences, 151, 90-100.

Lallemand, S., Heuret, A., Boutelier, D. (2005). On the relationships between slab dip, back‐arc stress, upper plate absolute motion, and crustal nature in subduction zones. Geochemistry, Geophysics, Geosystems, 6(9).

Leat, P.T., Larter, R.D. (2003). Intraoceanic subduction systems: introduction. Geological Society, London, Special Publications, 219(1), 1-17.

Li, C., Lu, Y., Wang, J. (2017) A global reference model of curie-point depths based on EMAG2. Sci. Rep. 7, 45129. <https://doi.org/10.1038/srep45129>

Li, H.-Y., Zhao, R.-P., Li, J., Tamura, Y., Spencer, C., Stern, R.J., Ryan, J.G., Xu, Y.-G. (2021). Molybdenum isotopes unmask slab dehydration and melting beneath the Mariana arc. Nature Communications, 12, 1-10.

Liu, Y. (2013). Numerical simulations on megathrust rupture stabilized under strong dilatancy strengthening in slow slip region. Geophys. Res. Lett., 40, 1311–1316,http://dx.doi.org/10.1002/grl.50298.

Maekawa H, Shozui M, Ishii T, Fryer P, Pearce JA. (1993). Blueschist metamorphism in an active subduction zone. Nature 364, 520–523.

Manea, V.C., Manea, M., Ferrari, L., Orozco-Esquivel, T., Valenzuela, R., Husker, A., Kostoglodov, V. (2017). A review of the geodynamic evolution of flat slab subduction in Mexico, Peru, and Chile. Tectonophysics 695, 27-52.

Matsumoto, T. (2007). Terrestrial heat flow distribution in Japan area based on the temperature logging in the borehole of NIED Hi-net, AGU Fall Meeting Abstracts, pp. T23A-1217.

McCaffrey, R., King, R.W., Payne, S.J., Lancaster, M. (2013). Active tectonics of northwestern U.S. inferred from GPS-derived surface velocities. J. Geophys.Res., 118, 709–723, http://dx.doi.org/10.1029/2012JB009473.

Müller, R.D., M. Sdrolias, C. Gaina, Roest, W.R. (2008). Age, spreading rates, and spreading asymmetry of the world's ocean crust, Geochem. Geophys. Geosyst., 9 (4), Q04006, doi:10.1029/2007GC001743.

Oakley, A.J., Taylor, B., Moore, G.F. (2008). Pacific Plate subduction beneath the central Mariana and Izu‐Bonin fore arcs: New insights from an old margin. Geochemistry, Geophysics, Geosystems, 9(6).

Ohara, Y., Reagan, M. K., Fujikura, K., Watanabe, H., Michibayashi, K., Ishii, T., ... Ribeiro, J. (2012). A serpentinite-hosted ecosystem in the Southern Mariana Forearc. Proceedings of the National Academy of Sciences, 109(8), 2831-2835.

Omori, S., Kita, S., Maruyama, S., Santosh, M. (2009). Pressure–temperature conditions of ongoing regional metamorphism beneath the Japanese Islands, Gondwana Research, 16, 458–469.

Pabst, S., Zack, T., Savov, I. P., Ludwig, T., Rost, D., Tonarini, S., Vicenzi, E. P. (2012). The fate of subducted oceanic slabs in the shallow mantle: Insights from boron isotopes and light element composition of metasomatized blueschists from the Mariana forearc. Lithos, 132, 162-179.

Peacock, S.M., Christensen, N.I., Bostock, M.G., Audet, P. (2011). High pore pressures and porosity at 35 km depth in the Mariana subduction zone, Geology, doi:10.1130/G31649.1.

Peltier, W.R. (2011). Mantle Viscosity. In: Gupta, H.K. (eds) Encyclopedia of Solid Earth Geophysics. Encyclopedia of Earth Sciences Series. Springer, Dordrecht, Holland. <https://doi.org/10.1007/978-90-481-8702-7_40>

Penniston-Dorland, S.C., Kohn, M.J., Manning, C.E. (2015). The global range of subduction zone thermal structures from exhumed blueschists and eclogites: Rocks are hotter than models. Earth and Planetary Science Letters 428, 243-254.

[Plümper](http://www.nature.com/ngeo/journal/v10/n2/full/ngeo2865.html#auth-1), O., [John](http://www.nature.com/ngeo/journal/v10/n2/full/ngeo2865.html#auth-2), T., [Podladchikov](http://www.nature.com/ngeo/journal/v10/n2/full/ngeo2865.html#auth-3), Y.Y., [Vrijmoed](http://www.nature.com/ngeo/journal/v10/n2/full/ngeo2865.html#auth-4), J.C., [Scambelluri](http://www.nature.com/ngeo/journal/v10/n2/full/ngeo2865.html#auth-5), M. (2017). Fluid escape from subduction zones controlled by channel-forming reactive porosity, Nat. Geoscience, 10, 150–156 (2017) doi:10.1038/ngeo2865

Pollack, H.N., Hurter, S.J. Johnson, J.R. (1993). Heat flow from the earth's interior: analysis of the global data set, Reviews of Geophysics, 31(3), 267-280.

Pozgay, S. H., Wiens, D. A., Conder, J. A., Shiobara, H., Sugioka, H. (2009). Seismic attenuation tomography of the Mariana subduction system: Implications for thermal structure, volatile distribution, and slow spreading dynamics. Geochemistry, Geophysics, Geosystems, 10(4).

Qu, R., Ji, Y., Zhu, W. (2021). Variations in wedge earthquake distribution along the strike underlain by thermally controlled hydrated megathrusts. Applied Sciences-Basel, 11, 7268. <https://doi.org/10.3390/app11167268>.

Qu, R., Ji, Y., Zhu, W., Zhao, Y., Zhu, Y. (2022). Fast and slow earthquakes in Alaska: Implications from a three-dimensional thermal regime and slab metamorphism, Applied Sciences-Basel, 12, 11139.

Ranero, C.R., Phipps Morgan, J., McIntosh, K., Reichert, C. (2003). Bending-related faulting and mantle serpentinization at the Middle America trench. Nature 425, 367-373.

Ribeiro, J.M., Stern, R.J., Kelley, K.A., Shaw, A.M., Martinez, F., Ohara, Y. (2015). Composition of the slab-derived fluids released beneath the Mariana forearc: Evidence for shallow dehydration of the subducting plate. Earth and Planetary Science Letters 418, 136-148.

Ruh, J.B., Le Pourhiet, L., Agard, P., Burov, E., Gerya, T. (2015). Tectonic slicing of subducting oceanic crust along plate interfaces: Numerical modeling. Geochemistry, Geophysics, Geosystems 16, 3505-3531.

Salisbury MH et al.2002. Proceedings of the ocean drilling program, initial reports, Leg 195. College Station, TX: Ocean Drilling Program.

Savov, I.P., Ryan, J.G., D'Antonio, M., Fryer, P. (2007). Shallow slab fluid release across and along the Mariana arc‐basin system: Insights from geochemistry of serpentinized peridotites from the Mariana fore arc. Journal of Geophysical Research: Solid Earth, 112(B9).

Seton, M., Muller, R.D., Zahirovic, S., Gaina, C., Torsvik, T.H., Shephard, G., Talsma, A., Gurnis, M., Turner, M., Maus, S., Chandler, M. (2012). Global continental and ocean basin reconstructions since 200 Ma. Earth Sci. Rev. 113 (34), 212–270. http://dx.doi. org/10.1016/j.earscirev.2012.03.002. (ISSN 0012-8252).

Siebert, L., Simkin, T., Kimberly, P. (2010). Volcanoes of the World, 3rd ed. Berkeley: University of California Press, 568.

Smith, W. H. F., Sandwell, D.T. (1997). Global seafloor topography from satellite altimetry and ship depth soundings, Science, 277, 1957–1962.

Stern, R. J., Hargrove, U.S. (2003). The Anatahan Felsic Province in the mariana arc system. AGUFM, 2003, V32B-1010.

Stern, R., Smoot, N.C. (1998). A bathymetric overview of the Mariana forearc. Island Arc, 7(3), 525-540.

Stern, R. J., Reagan, M., Ishizuka, O., Ohara, Y., Whattam, S. (2012). To understand subduction initiation, study forearc crust: To understand forearc crust, study ophiolites. Lithosphere, 4(6), 469-483.

Stern, R. J., Tamura, Y., Masuda, H., Fryer, P., Martinez, F., Ishizuka, O., Bloomer, S.H. (2013). How the Mariana Volcanic Arc ends in the south. Island Arc, 22(1), 133-148.

Stolper, E., Newman, S. (1994). The role of water in the petrogenesis of Mariana trough magmas. Earth and Planetary Science Letters, 121(3-4), 293-325.

Suenaga, N., Ji, Y., Yoshioka, S., Feng, D. (2018). Subduction thermal regime, slab dehydration, and seismicity distribution beneath Hikurangi based on 3D simulations. J. Geophys. Res. solid earth, 123. <https://doi.org/10.1002/2017JB015382>.

Suenaga, N., Yoshioka, S. Ji, Y. (2021). 3-D thermal regime and dehydration processes around the regions of slow earthquakes along the Ryukyu Trench. Sci Rep 11, 11251. https://doi.org/10.1038/s41598-021-90199-2

Syracuse, E.M., van Keken, P.E., Abers, G.A. (2010). The global range of subduction zone thermal models. Physics of the Earth and Planetary Interiors 183, 73-90.

Tackley, P.J., Xie, S. (2003). Stag3D: A code for modeling thermochemical multiphase convection in Earth's mantle, Computational Fluid and Solid Mechanics 2003, edited by K. J. Bathe, pp. 1524-1527, Elsevier B.V., Amsterdam, Netherlands.

Tamblyn, R., Zack, T., Schmitt, A., Hand, M., Kelsey, D., Morrissey, L., Pabst, S., Savov, I. (2019). Blueschist from the Mariana forearc records long-lived residence of material in the subduction channel. Earth and Planetary Science Letters 519, 171-181.

Tanaka, A. (2004). Geothermal gradient and heat flow data in and around Japan (II) Crustal thermal structure and its relationship to seismogenic layer. Earth, planets and space 56, 1195-1199.

Tanaka, A., Yamano, M., Yano, Y., Sasada, M. (2004). Geothermal gradient and heat flow data in and around Japan (I): Appraisal of heat flow from geothermal gradient data. Earth, planets and space 56, 1191-1194.

Trabant, C., Hutko, A.R., Bahavar, M., Karstens, R., Ahern, T., Aster, R. (2012). Data Products at the IRIS DMC: Stepping Stones for Research and Other Applications, Seismol. Res. Lett., 83(5), 846-854, <https://doi.org/10.1785/0220120032>.

van der Hilst, R., Seno, T. (1993). Effects of relative plate motion on the deep structure and penetration depth of slabs below the Izu-Bonin and Mariana Island arcs. Earth and Planetary Science Letters, 120(3-4), 395-407.

van Keken, P.E. (2003). The structure and dynamics of the mantle wedge. Earth and planetary science letters, 215(3-4), 323-338.

van Keken, P.E., Hacker, B. R., Syracuse, E. M., Abers, G. A. (2011). Subduction factory: 4. Depth‐dependent flux of H_2_O from subducting slabs worldwide. Journal of Geophysical Research: Solid Earth, 116(B1).

van Keken, P.E., Kiefer, B., Peacock, S.M. (2002). High-resolution models of subduction zones: Implications for mineral dehydration reactions and the transport of water into the deep mantle. Geochemistry, Geophysics, Geosystems 3, 1.

Wada, I., Wang, K. (2009). Common depth of slab-mantle decoupling: Reconciling diversity and uniformity of subduction zones. Geochemistry, Geophysics, Geosystems 10.

Wada, I., Wang, K., He, J., Hyndman, R.D., (2008). Weakening of the subduction interface and its effects on surface heat flow, slab dehydration, and mantle wedge serpentinization, J. Geophys. Res., 113 (B04402), doi:10.1029/2007JB005190.

Wang, K., [Tréhu](http://www.sciencedirect.com/science/article/pii/S026437071530017X)[,](http://www.sciencedirect.com/science/article/pii/S026437071530017X#aff0010) A.M. (2016). Some outstanding issues in the study of great megathrust earthquakes-The Mariana example, [J. Geodyn.](http://www.sciencedirect.com/science/journal/02643707), 98, 1–18.

Wessel, P., Smith, W.H.F. (1998). New improved version of the generic mapping tools released, Eos Trans. Am. Geophys. Union, 79, 579.

Watts, A.B. (2001). Isostasy and Flexure of the Lithosphere. Cambridge University Press.

Yabe, S., Ide, S., Yoshioka, S. (2014). Along-strike variations in temperature and tectonic tremor activity along the Hikurangi subduction zone, New Zealand. Earth, Planets and Space, 66(1), 1-15.

Zhang, J., Zhang, F., Lin, J., Yang, H. (2021). Yield failure of the subducting plate at the Mariana Trench. Tectonophysics 814, 228944.

Zheng, Y.F., Chen, Y.X. (2016). Continental versus oceanic subduction zones. National Science Review 3, 495-519.

Zhu, W., Ji, Y., Qu, R., Xie, C., Yoshioka, S., Zeng, D. (2022). Thermal regime and slab dehydration beneath the Izu‐Bonin arc: Implications for fast and slow subduction earthquakes. Terra Nova 34, 103-112.
